# Powered Two-Wheeler Riding Profile Clustering for an In-Depth Study of Bend-Taking Practices

**DOI:** 10.3390/s20226696

**Published:** 2020-11-23

**Authors:** Mohamed Diop, Abderrahmane Boubezoul, Latifa Oukhellou, Stéphane Espié

**Affiliations:** 1 TS2-SIMU&MOTO, Université Gustave Eiffel, IFSTTAR, F-77447 Marne-la-Vallée, France; mohamed.diop@univ-eiffel.fr (M.D.); stephane.espie@univ-eiffel.fr (S.E.); 2 COSYS-GRETTIA, Université Gustave Eiffel, IFSTTAR, F-77447 Marne-la-Vallée, France; latifa.oukhellou@univ-eiffel.fr

**Keywords:** powered two-wheeler, riding profiles, riding activity analysis, data mining, wearable sensors

## Abstract

The understanding of rider/vehicle interaction modalities remains an issue, specifically in the case of bend-taking. This difficulty results both from the lack of adequate instrumentation to conduct this type of study and from the variety of practices of this population of road users. Riders have numerous explanations of strategies for controlling their motorcycles when taking bends. The objective of this paper is to develop a data-driven methodology in order to identify typical riding behaviors in bends by using clustering methods. The real dataset used for the experiments is collected within the VIROLO++ collaborative project to improve the knowledge of actual PTW riding practices, especially during bend taking, by collecting real data on this riding situation, including data on PTW dynamics (velocity, normal acceleration, and jerk), position on the road (road curvature), and handlebar actions (handlebar steering angle). A detailed analysis of the results is provided for both the Anderson–Darling test and clustering steps. Moreover, the clustering results are compared with the subjective data of subjects to highlight and contextualize typical riding tendencies. Finally, we perform an in-depth analysis of the bend-taking practices of one subject to highlight the differences between different methods of controlling the motorcycle (steering handlebar vs. rider’s lean) using the rider action measurements made by pressure sensors.

## 1. Introduction

In recent years, increasingly numerous cities in France and elsewhere in Europe have been experimenting with or setting up car-free zones. The objective is to limit the usage of private cars. Consequently, powered two-wheeled vehicles (PTWs) are becoming increasingly popular. In France, PTW users are particularly exposed to road accidents and fatalities. According to the French Observatory of Road Safety (ONISR) [1], PTWs barely constitute 2% of road traffic, but they account for 23% of road deaths and 44% of serious accidents, and PTW users are 18 times more likely to be killed than a car driver when normalizing for the number of kilometers traveled. In 2017, in France, 32% and 39% of fatal accidents of mopeds and motorcycles, respectively, occurred without an identified third party. In particular, loss of control in bends accounts for more than 42% of single-vehicle accidents. An understanding of the bend-taking behavior plays a significant role in scientific knowledge improvement and road safety. The in-depth analysis of rider/PTW interaction modalities remains an open question.

The literature on motorcycle riding study can be categorized into two categories: the first category addresses the rider’s behavior analysis, and the second addresses rider modeling. A general overview of this categorization is given in [2,3]. In the first category, the most important topics addressed are the handlebar control (steering torque versus steering angle), dominance of different types of control (handlebars versus rider lean) [4,5], and differences between experienced and novice riders [6]. More  recently, in [7,8] the authors addressed the problem of motorcycle rider model identification based on experimental data recorded during road tests with a fully instrumented motorcycle.

The main scientific challenge to overcome is to design a model of steering control most similar to that achieved by PTW riders. To achieve this aim, it is essential to represent the sensorimotor processes used by a rider to control the vehicle trajectory through different modalities of actions on the vehicle at an appropriate level of complexity [9]. From a technical standpoint, the question relates to the embedded measurement of the rider/PTW interactions and how to capture these interactions. Accordingly, an experimental study has been carried out by using both pressure sensors and pressure gauges. Pressures have been recorded at the following contact points: left and right handlebar, saddle, tank, and footrests. These measures have been added to those related to vehicle dynamics.

After collecting all of these data, we address the following scientific question in this ongoing research work; Can the rider’s behavior be reduced to a rider “profile” or categorized within classes? We postulate that the rider can be “reduced” to a psychometric problem and that these different strategies should be consequently identified using discriminative behavioral markers.

Various research works in the scientific literature focus on driving behavior study and driving style characterization. The approaches based on machine learning techniques are widely used for this purpose. The driver’s behavior is analyzed using the signals issued from real experiments. Based on the features extracted from signals, the proposed approaches aim at clustering the subjects into different behavioral groups [10,11,12]. In this paper, we follow this line of research to study the global behavior of motorcyclists.

In comparison with the existing literature about driver profiles [13,14], research into PTW rider behavior based on real bend-taking manoeuvres is limited. Some research has been undertaken on simulators; in this regard, we can cite the works in [15,16].

In this paper, as a first step in riding profile classification “recognition”, exploratory data analysis is chosen, which thus permits us to analyze, discover, and potentially prove certain hypotheses about real riding practices. This approach will help us provide solutions to the following problems; how to visualize the rapid and robust classification based on the quantitative variables, how to combine the classification produced based on quantitative data with the remaining qualitative variables to interpret the classification, and how to provide evidence for the most important explanatory variables for each identified class.

The approach presented in this paper is an easy-to-use methodology based on data mining approaches suitable for the study of the riders’ behavior and for the understanding of the way they interact with their vehicle when negotiating bends. In this study, we are trying to fill the knowledge gap on how a range of riders approach bends in real life. The intra- and inter-variability of riders, the particular dynamics of these vehicles, and the tight coupling between the rider and the vehicle make the problem particularly complex. The main contributions of this paper are summarized in the following.

- We propose an easy-to-use methodology for providing data analysis tools to road safety researchers to help them in their study of the motorcyclists’ behavior during bend negotiation maneuver.
- We successfully apply the proposed framework to a real dataset of sensor data collected during experimentation that involves eight subjects with different profiles.
- We present a multi-sensors architecture to capture the rider’s actions during the bend taking maneuver, and we analyze with a certain level of detail the collected data, which makes this manuscript a valuable reference for practitioners interested in such topic of research.

The human riding behavior is a complex concept, and its characterization may lead to a better understanding of the rider’s decisions when encountering different situations. This characterization will prevent collisions and design the riding models, which is one of the core algorithms that might make the future of self-riding motorbikes possible. Autonomous vehicles have to interact with other vehicles (even non-autonomous ones), and understanding their driving style can provide valuable information to avoid traffic collisions.

The paper is organized as follows. Section 2 addresses the experimental protocol. Section 3 describes the proposed methodology for PTW rider clustering. The results and discussion of the clustering step are presented in Section 4, and an in-depth analysis of riding control of a selected subject is given in Section 5. The conclusion and perspectives are provided in Section 6.

## 2. Experimental Protocol: Data Collection And Description

**Figure 1:**
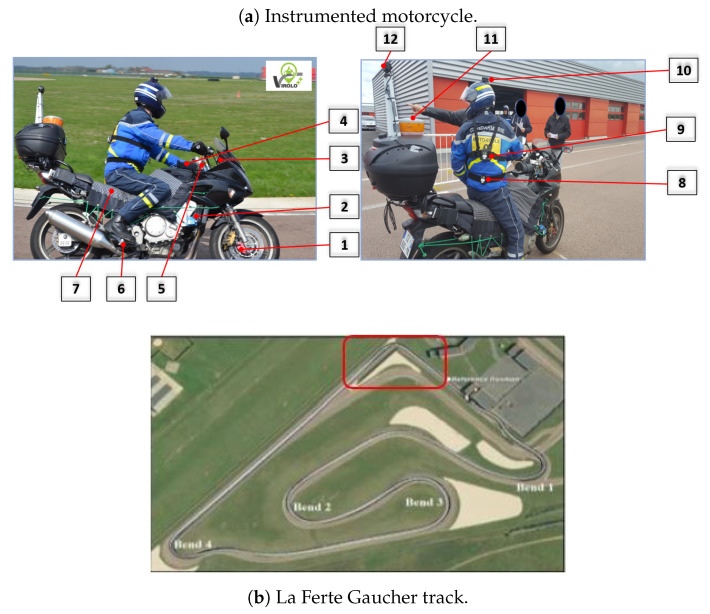
The heavily instrumented motorcycle and the La Ferte Gaucher track.

The data used in this paper come from a real-life cornering experiment using a heavily instrumented motorcycle (cf. Figure 1a) on the La Ferte Gaucher track (cf. Figure 1b) [17].

### 2.1. Experimental Protocol

**Figure 2:**
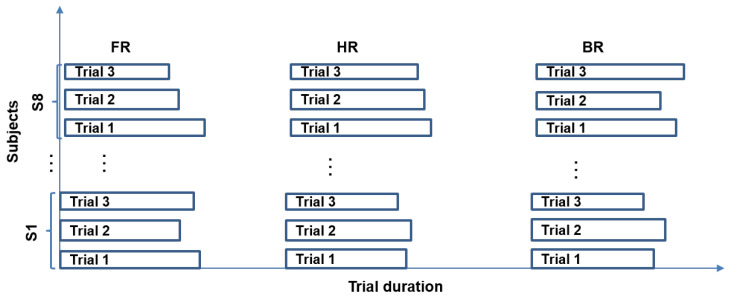
Three riding trials by eight subjects with each riding instruction: FR, HR, and BR.

The La Ferte Gaucher circuit track in France was chosen for the experimental protocol. The main objective of this experimental protocol is to use sensors to collect data relating to the natural, handlebar, and body riding during bend-taking. Sensors and their description are summarized in Table 1. It is hypothesized that some riders approach a bend using the handlebars more than the body, while others do the opposite. Eight subjects participated in the experiment by undertaking three trials with each of the following instructions; free riding (FR), riding principally using body movements (BR), and riding principally using the handlebar (HR). In BR, the subjects use their body movements (foot, buttocks, knees) as much as possible, as opposed to HR, where they use more of the handlebar while negotiating bends. In FR, the subjects ride in their usual manner. Because of the velocity, the duration of the trials may differ for the same subject for a given riding instruction. Similarly, this difference is observed among the subjects for all riding instructions (cf. Figure 2).

### 2.2. Data Collection

To collect data related to motorcycle riding, several sensors have been placed at strategic points on the motorcycle. As the data come from several sensors, an on-board computer is used for data centralization. For each riding instruction, the data relating to the motorcycle’s dynamics, rider’s actions, and their interactions are collected for all bends of the track. However, in this paper, we only use the data relative to the bend highlighted in red (cf. Figure 1b). Information about the eight subjects in the experiment is provided in Table 2. LA indicates the age of licenses, and km is the number of kilometers traveled on PTWs during the year preceding the experiment. The preference and instruction order designate the marks (out of ten) given to HR, the preference between BR and HR, and the instruction order.

**Figure 3:**
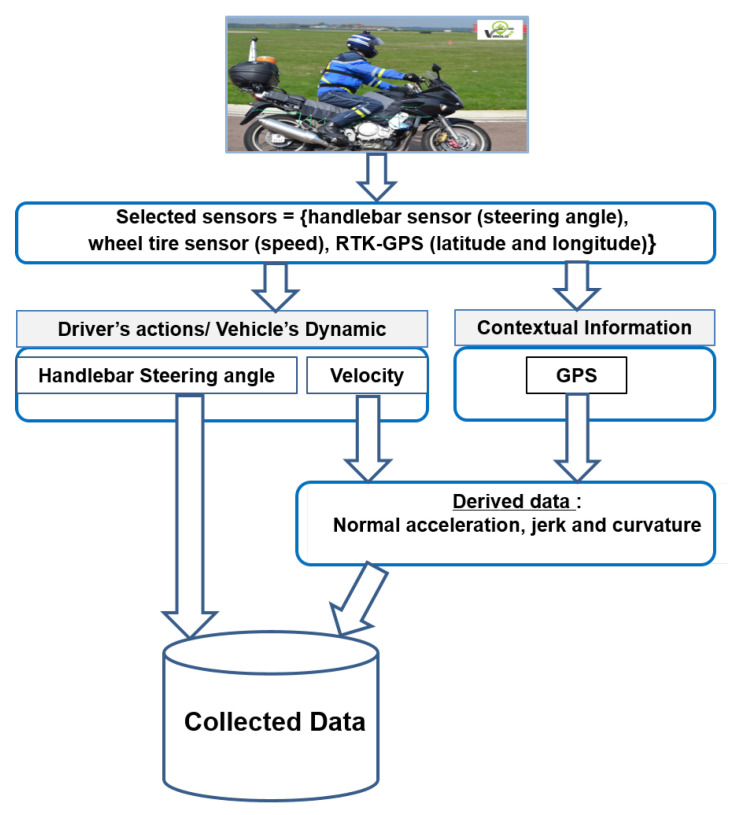
Data collection process.

Among the multiple signals collected on the bend of interest, we only focus on the measurements of the handlebar steering angle (δ), velocity (v), and GPS positions (longitude and latitude). In addition, derived signals such as the track curvature (c), normal acceleration (an), and jerk (J) are calculated using the velocity and GPS positions, see Figure 3.

### 2.3. Data Representation

The final dataset that we obtain at the end of the experimentation is composed of δ, v, an, J, and C. For this dataset, we use the following notations,

$$D={\langle D^{l}\rangle }_{l\in \{FR,HR,BR\}},$$

with $D^{l}={\left(x_{k}\right)}_{\left\{k=1,\ldots ,N\right\}},$ where *N* is the total number of sequences of riding instruction *l* in {FR,HR,BR}. Each sequence $x_{k}={\left(x_{k,m}\right)}_{m=1,\ldots ,M}$ is a set of values observed over a period of time ${\left(t_{m}\right)}_{m=1,\ldots ,M}$, M (an integer between 82 and 124) is the total number of observations, and $x_{k,m}$ is given by

$$x_{k,m}=\left\{\delta_{k,m},v_{k,m},{an}_{k,m},J_{k,m},C_{k,m}\right\},$$

where $\delta_{k,m}$, $v_{k,m}$, ${an}_{k,m}$, $J_{k,m}$ and $C_{k,m}$ are the handlebar steering angle in degrees; velocity in m/s; normal acceleration in $m/s^{2}$; jerk, which is the third derivative of velocity in $m/s^{3}$; and curvature in $m^{-1}$ measured at time $t_{m}$ for sequence *k*, respectively.

## 3. Clustering Methodology

In this section, the clustering methodology for 8 subjects with respect to the parameters δ, v, an, J, and C is described. A GPS-based approach for the behavior of PTW riders in bends [25] is used to select the curvature parameter, while the remaining parameters are chosen based on [26].

**Figure 4:**
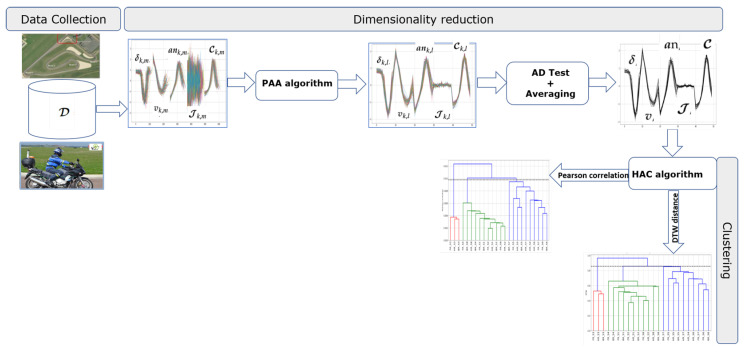
Overview of the clustering methodology.

Three steps characterize our proposed methodology (see Figure 4): (1) Dimensionality reduction to extract a reduced number of descriptors, (2) Anderson–Darling test for the homogeneity study of the three trials, and (3) clustering of subjects.

### 3.1. Dimensionality Reduction Using the Piecewise Aggregate Approximation (PAA) Algorithm

**Figure 5:**
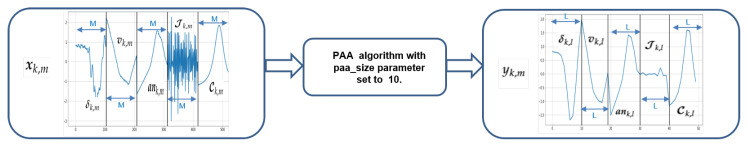
An example of the PAA algorithm applied on $x_{k,m}$.

The hierarchical agglomerative clustering (HAC) algorithm that we use in this paper is parameterized with metrics measuring the similarity between two samples. In this situation, as mentioned in [27], the most promising solutions involve first performing a dimensionality reduction on the data. In [27], the authors theoretically and empirically show that the PAA algorithm is superior to other dimensionality reduction techniques such as the singular value decomposition (SVD), discrete Fourier transform (DFT), and discrete wavelet transform (DWT) algorithms. Then, we use the PAA algorithm on each z-normalized $x_{k,m}$ as suggested in [27]. The main idea of the PAA algorithm is to reduce the input z-normalized $x_{k,m}$ dimensionality by splitting it into equally sized segments (PAA-size) and averaging the values of points in each segment [28]. In our case, the PAA-size parameter is set to 10 so that we obtain approximately 10 samples in each segment per second, which coincides with the frequency of our data (10 Hz). After applying the PAA algorithm to each riding variable of a given set $x_{k,m}$, we obtain the reduced data $y_{k,l}$ with l=1,…,L with L=10. An example of the PAA algorithm applied on our data is given in Figure 5.

### 3.2. Homogeneity Study for the Three Attempts of Each Instruction Using the Anderson–Darling Test

Because the subjects were not used to riding the instrumented motorcycle, δ, v, an, J, and C may differ from one trial to another. Statistical tests such as the Kolmogorov–Smirnov [29] or Anderson–Darling [30] tests can be used to check this assumption. However, for small samples, the Anderson–Darling test is more powerful than the Kolmogorov–Smirnov test [31,32]. Thus, we use Anderson–Darling (AD) test, which states under its null hypothesis that the samples are drawn from the same distribution.

In our case, the AD test is performed on $y_{k,m}$ obtained after performing the PAA dimensionality reduction. Therefore, for each instruction and a given riding variable, we can test whether there is a significant difference among the distributions of the three trials. In the Anderson–Darling test, we reject the null hypothesis if the probability value (*p*-value) is less than the reference threshold α, which is usually fixed at 5%. In other words, the *p*-value is the greatest threshold for which the null hypothesis is accepted.

Table 3 provides the Anderson–Darling test *p*-value for all riding instructions and variables of 8 subjects. In this table, the minimum value, which is shown in bold, is 0.24, which is greater than α (0.05). Therefore, for all riding instructions and variables, we cannot reject the null hypothesis. Thus, the existing difference among the three trials is not significant for a given subject in all riding instructions and variables.

**Figure 6:**
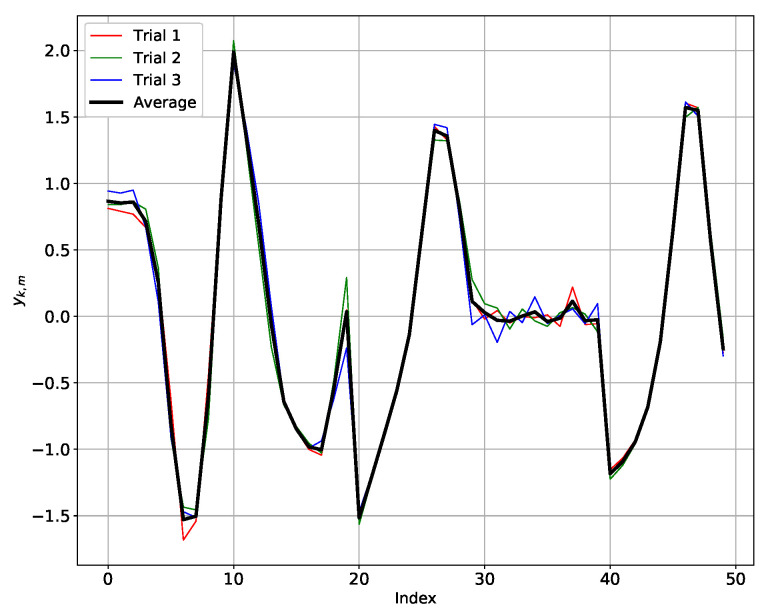
Average of three trials of subject S1 in the FR instruction.

In conclusion, the AD test results enable us to reduce the three trials in each riding instruction by taking their average. Thus, for a given riding instruction, we can characterize each subject by an average behavior during bend taking, as shown in Figure 6. For the eight subjects and three riding instructions, we obtain a dataset in a matrix of size 24×50. The number of rows 24 is obtained by multiplying the average behavior of the 8 subjects by the number of riding instructions, and the number pf columns 50 is obtained by multiplying L=10 the window size chosen in the PAA algorithm by the number of features which is equal to 5.

### 3.3. Clustering of Subjects Using Hierarchical Agglomerative Clustering (HAC)

The final step of the methodology consists of using hierarchical agglomerative clustering (HAC) [33] on the data matrix $Y_{ij}$ composed by the average behaviors of the subjects during the three riding instructions. To use this algorithm, two methods must be implemented to calculate the distance between two observations and the distance between two clusters (linkage criterion). To calculate the distance between two observations $Y_{ij}$ and $Y_{mj}$ with fixed *i*, *m*, where i≠m, we use Pearson correlation coefficient [34] and Dynamic Time Warping (DTW) [35] as metrics. Following are the main steps of HAC applied in our study (see Algorithm 1): 

**Algorithm 1** The hierarchical agglomerative clustering (HAC) algorithm applied on the $Y_{ij}$ data matrix.**Require:**$Y_{ij}$ data matrix. 1:  Calculate the similarity: using the Pearson correlation and DTW metrics between each row pair of the data matrix $Y_{ij}$. 2:  Compute the proximity matrix. 3:  Merge the closest clusters according to the proximity matrix. 4:  Update the proximity between new and original clusters. 5:  Repeat steps 3 and 4 until a single cluster is obtained.

The distance between two clusters is measured by the single linkage criterion, which is the minimum distance among cluster data points.

## 4. Results and Discussion

In this section, we present the results obtained from the HAC algorithm. Choosing the number of clusters is the main difficulty for any clustering algorithm and has always been a subject of discussion, particularly with respect to unsupervised automatic clustering methods. Several criteria have been proposed in the literature, such as the Bayesian information criterion (BIC) [36], elbow rule, and gap statistic [37]. However, in our case, the number of clusters is chosen in relation to the experiment. If each subject respects the driving instructions while being consistent with their statements of riding preference (HR or BR), the ideal number of clusters is 2.

**Figure 7:**
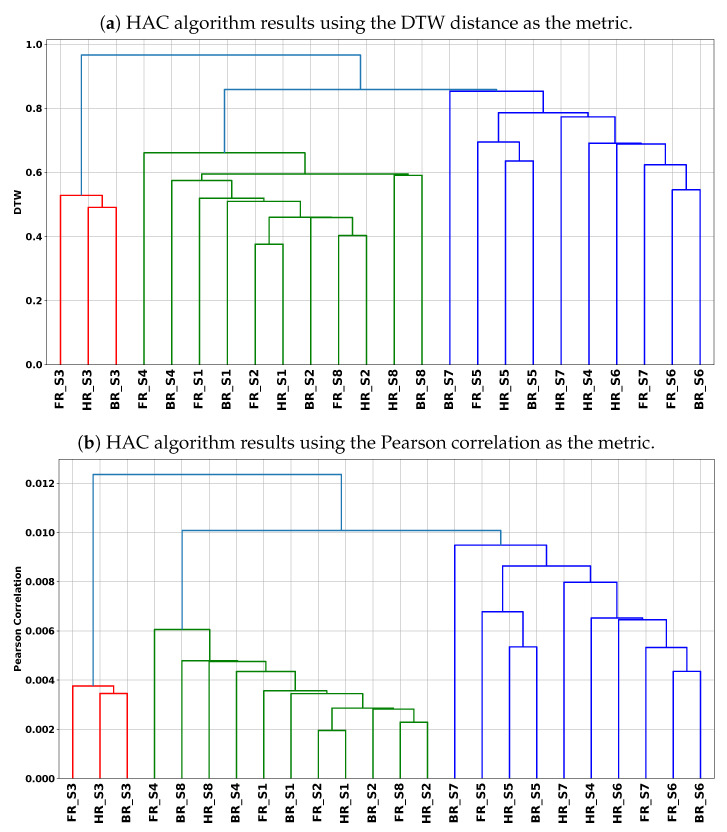
Clustering results of the HAC algorithm with the Dynamic Time Warping (DTW) and Pearson correlation metrics.

Figure 7 shows the dendrogram of the HAC results using the DTW and Pearson correlation metrics. The first analysis of the dendrograms shows that a clustering with 2 clusters is not a good idea. Indeed, the analysis will compare subject S3 (FR, HR, and BR) against the remaining subjects. Thus, we cut the dendrograms to have 3 clusters, as listed in Table 4. This table shows that we obtain the same results for both the DTW and Pearson correlation metrics.

### 4.1. Clusters Analysis

In this paragraph, we conduct cross-referencing for the analysis results of riding behavior extracted from the data mining (cf. Table 4) and stated riding preference (handlebar steering or body movements) from Table 2. This cross-analysis enables us to determine the extent to which the subjects comply with the riding instructions.

- Cluster 1 analysis: This cluster is essentially composed of three riding instructions of subject S3. An in-depth analysis of the dendrograms shows that in this cluster, the similarity is more observed between HR and BR instructions. Therefore, subject S3 has difficulty in differentiating between HR and BR instructions. Thus, for this subject, contradictions can be observed in terms of his riding preference (BR) and compliance with the HR and BR instructions.
- Cluster 2 analysis: Subjects S2, S4, and S8 have their riding instructions in this cluster, whereas subject S4 has only FR and BR instructions. Therefore, subjects S2, S4, and S8 did not respect the HR and BR riding instructions. Subject S4, who declared a preference for BR instruction, is consistent with his declaration of preference while respecting the riding instructions.
- Cluster 3 analysis: For the same reasons mentioned in the analysis of Cluster 2, subjects S5, S6, and S7 did not respect the HR and BR riding instructions. Subject S4, who has his HR instruction in this cluster, appears to respect this riding instruction.

In this cross-referencing, we detect an atypical rider, namely, subject S3, who behaves very differently from the other riders. This behavior is justified by the fact that this subject is obtaining a motorcycle license (LA is 0). Subject S4 appears to be the only subject to comply with the HR and BR riding instructions. Based on this hypothesis, we say that cluster 2 is composed of subjects using the body more than the handlebars, while cluster 3 is composed of subjects using the handlebars more than the body.

### 4.2. Interpretation of the Clustering Results

**Figure 8:**
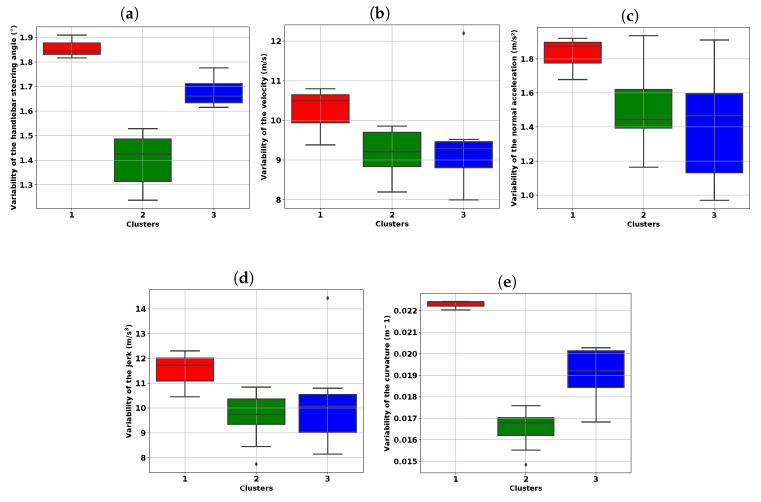
Distribution of the standard deviation by cluster for the five riding variables: (**a**) Handlebar steering angle, (**b**) Velocity, (**c**) Normal acceleration, (**d**) Jerk, and (**e**) Curvature.

The riders who likely have a preference for handlebar riding exhibit significant variability of the handlebar steering angle. Similarly, riders with less variability in the handlebar steering angle are potential candidates to prefer riding with body control. To measure the variability of the handlebar steering angle, we use the standard deviation, which is a good and simple indicator. We use the average signal of three trials of our raw data (cf. Figure 2) to calculate the standard deviation. Figure 8a shows that the variability of the handlebar steering angle contributes the most to distinguishing the two main clusters (2 and 3). To verify this observation, we perform the AD-test between the standard deviation distributions of clusters 2 and 3. For the handlebar steering angle and curvature variables, we reject the null hypothesis at the threshold α=5%. Thus, the existing difference between clusters 2 and 3 is significant for the variability of the handlebar steering angle and curvature variables, as depicted in Table 5.

The elements in cluster 2 have low variability of the handlebar steering angle compared those in cluster 3. Thus, cluster 2 gathers the elements riding with the body, while those of cluster 3 ride with the handlebars. Therefore, subjects S1, S2, and S8 are in contradiction with their riding preference, while subjects S5, S6, and S7 comply with their statements. This hypothesis does not contradict the behavior of subject S4, who appeared to be the only one to comply with the HR and BR instructions.

Based on the results in this section, we conclude that our initial hypothesis has been verified: the driving profile resulting from the interaction between the rider-vehicles-infrastructure can be categorized into classes. These classes represent different riding strategies, namely, handlebar riding and body riding. These different strategies have been identified using discriminative behavioral markers, which were calculated based on data obtained from physical sensors such as the handlebar steering angle and velocity as well as calculated features such as the normal acceleration, jerk, and curvature. Our explanation is that through this characterization of the driving profile, we can capture the sensorimotor processes used by the central nervous system of a rider to control the vehicle: visual anticipation, vestibular perception of the gravito-inertial forces, management of the handlebars, and postural adaptations required to control the vehicle tilt. These sensorimotor processes may differ from one rider to another. This difference is mainly related to her/his riding experience. Nevertheless, at a certain level, there is a common behavior. Another issue can arise from this data analysis: Is there a difference between methods of controlling the motorcycle (steering handlebar vs. rider’s lean) in the bend-taking maneuver? In the following section, we will highlight these differences by performing an in-depth analysis of subject S4 by using rider action measurements made by pressure sensors.

## 5. In-Depth Analysis of the Behavior of Subject S4

**Figure 9:**
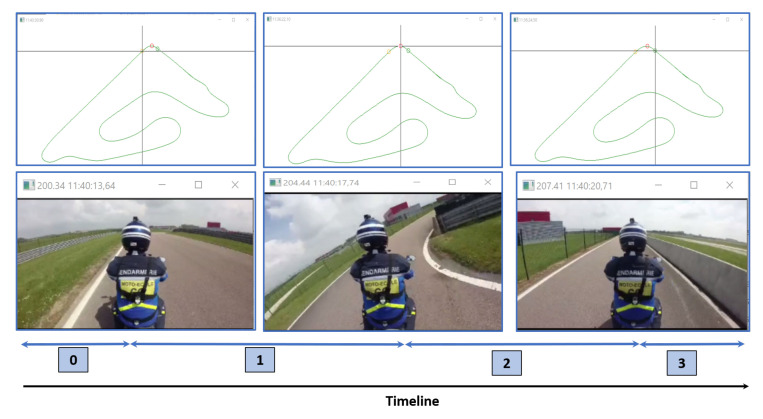
Average of three trials of subject S4 in the FR instruction. The curve maneuver is divided into four phases: Phase 0, Phase 1, Phase 2, and Phase 3.

In the case of PTWs, the driver is heavily involved in the driving task. The driver is continuously trying to ensure the stability of his/her vehicle to counter the gyroscopic effect, which plays an essential role in motorcycle driving, particularly during a turn [38]. For cornering, we note a significant variability in the real practices of drivers. Taking a curve requires the PTW driver to tilt the vehicle to compensate for the inertia caused by a change in direction. To perform this action, it is necessary to produce a tilt on the roll axis towards the side that one wants to approach during the change of direction. This change in direction may be initiated depending on the practices of each driver. The methods to control the motorcycle during a turn can be categorized into two main categories. In the first category, the rider applies a force on the handlebar, which results in a moment around the steering axis, i.e., steering torque. In the second category, the rider applies a moment directly in the roll direction by basically changing his/her center of gravity in the lateral direction. This shift in body weight results in action and reaction forces in the vertical direction on the foot-pegs and saddle. The present work aims to analyze and compare different motorcycle riders in operating the motorcycle and especially how they initiate a curve in terms of actions measured by pressure sensors. The previous data analysis was performed based on the dynamical behavior of the motorcycle. To perform this in-depth analysis, each maneuver is manually labeled using proprietary software, which is composed of two synchronized applications: one can browse the dynamical data, and the other browses the video recording. The cornering maneuver is divided into four phases (see Figure 9):

- Phase 0 corresponds to when the rider travels in a straight line before entering the curve.
- Phase 1 corresponds to the time when the rider initiates the curve and reaches the middle of the curve. In the following figures, the time interval corresponding to phase 1 is represented by a shaded area.
- Phase 2 corresponds to the elapsed time from the moment that the rider reaches the middle of the curve and that when he/she exits the turn.
- Phase 3 corresponds to when the rider is traveling in a straight line after exiting the curve.

**Figure 10:**
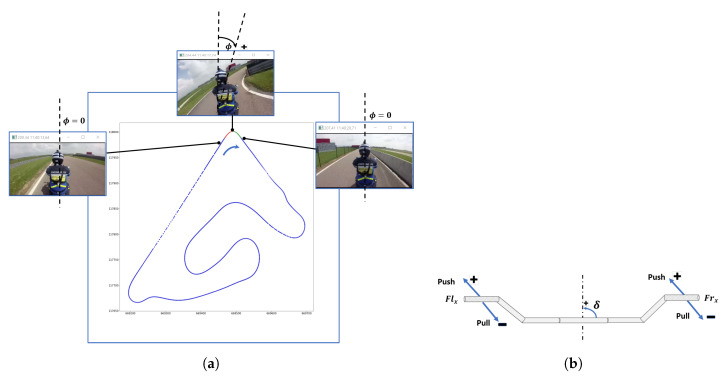
(**a**) Roll (ϕ) and (**b**) steering (δ) angles and forces $\left(fl_{x},fr_{x}\right)$ applied on the handlebar steering conventions.

Before beginning our in-depth study, we will present the conventions adopted in this work regarding the orientation of roll angles, steering angles, and forces applied on the handlebar steering, see Figure 10a,b.

**Figure 11:**
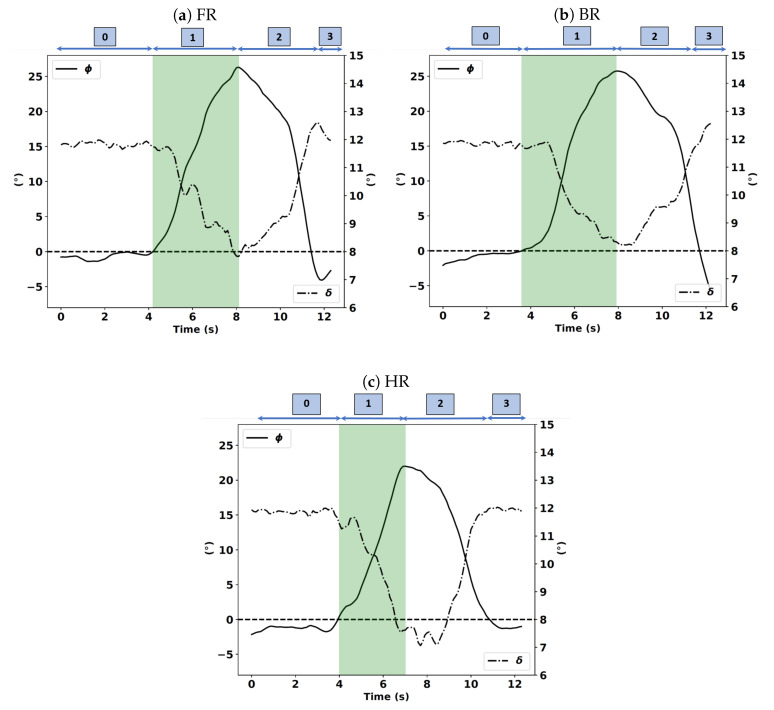
Handlebar steering angle δ (-.) and roll angle ϕ(-) expressed in degrees (${}^{{}^{\circ}}$) of subject S4 for the FR, BR, and HR instructions.

Rider “S4” is used to carry out an in-depth analysis of the turn maneuver with three instructions, namely, free riding (FR), riding principally using body movements (BR), and riding principally using the handlebar (HR). The previous data analysis study shows that this rider complied with the given instructions. We also find that the FR instruction complies with the BR instruction, which is coherent with the rider’s declared preference. This in-depth analysis of how rider S4 operates a motorcycle based on the average behavior made in the three trials of subject S4 with three instructions: HR, BR, and FR. Therefore, in this in-depth analysis, we expect to find that this rider will initiate the curve through a torque on the handlebars in the case of HR instruction. We do not have a sensor that measures the force-torque applied by the rider on the handlebars; instead, we have sensors that measure the forces applied by the rider on the right handlebar and left handlebar halves, knowing that the torque is the result of the application of the latter. In free riding and body riding cases, the rider will initiate the turn by the movement of his/her body by either moving her/his center of gravity or pressing on the footrest. The next figure shows the handlebar steering and roll angles of the average behavior of subject S4 during the riding instructions, see Figure 11a–c.

**Figure 12:**
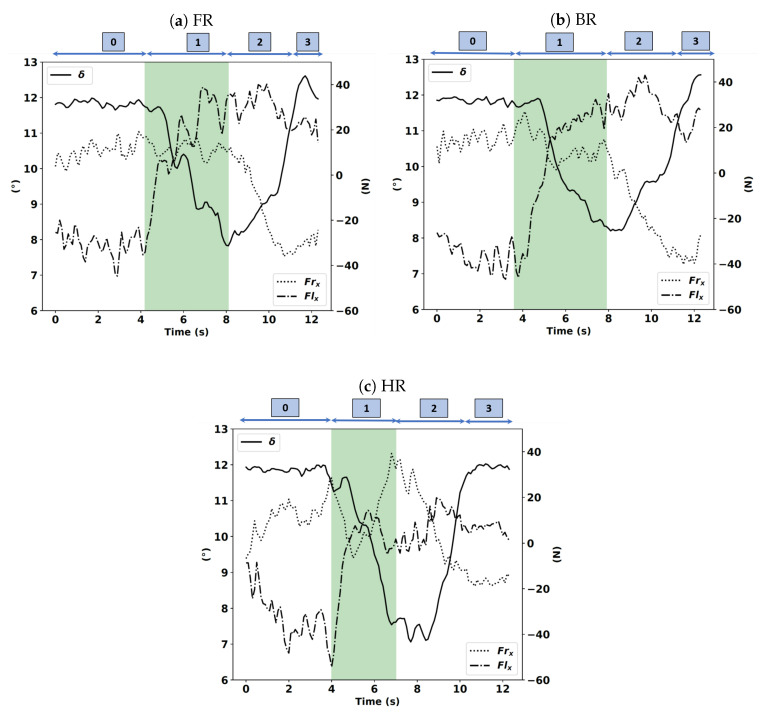
Applied forces on the left (Flx) and right (Frx) handlebars along the longitudinal axis expressed in newtons (N), and the handlebar steering angle (δ) expressed in newtons (${}^{{}^{\circ}}$) in the FR, BR, and HR instructions.

In phase 0, the roll angle is approximately equal to 0, which implies that the motorcycle is upward. When phase 1 begins, the motorcycle starts to roll in the direction of the corner. The roll angle slowly increases until it reaches its maximum, which corresponds to the middle of the curve; this corresponds to when phase 1 ends and phase 2 begins. From this moment, the centrifugal force becomes too large and forces the motorcycle to roll back until the motorcycle returns to the upward position. In the handlebar riding instruction case (see Figure 12c), we notice that the steering angle starts decreasing before the beginning of phase 1, which corresponds to the curve initiation time, not the other instructions; the steering angle remains constant. In that case, we can hypothesize that by applying a steering torque, the rider initiates the curve. The rider creates this torque when he/she applies forces on the handlebar along the longitudinal axis, namely, $Fr_{x}$ and $Fl_{x}$. As depicted in Figure 12a–c, only in the HR case is $Fr_{x}$ significantly decreasing at the beginning of phase 1, which corresponds to the curve initiation.

**Figure 13:**
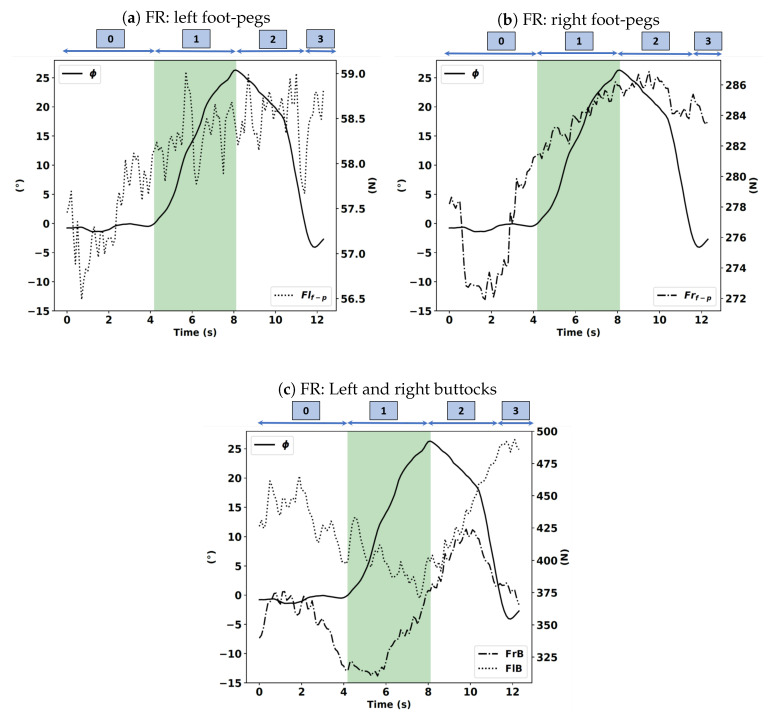
Left and right foot-pegs ($Fl_{f-p}$, $Fr_{f-p}$), buttocks forces (FlB, FrB) expressed in newtons (N) with the roll angle (ϕ) expressed in degrees (${}^{{}^{\circ}}$) in the FR instruction.

Note that this turn is a right turn. Therefore, if the driver tries to initiate the corner with his body and not through the handlebars, he/she will either move his center of gravity or press on the right footrest. We will focus on the actions of the driver immediately before the start of phase 1. As shown in Figure 13a–c, there is a change in value of the force applied by the driver on the right footrest during the FR case. The change is approximately 274 N, which corresponds to t = 2 s. From this instant, this force continuously increases until the end of phase 1 and the beginning of phase 2, where it reaches the value of 286 N. Meanwhile, there is no significant variation in the force applied by the driver on the left footrest. We notice the same behavior on the force applied by the right buttock, which means that simultaneously, the rider is trying to move his center of gravity and pressing the right footrest. This observation can be explained by the fact that the turn in this study is a right turn.

**Figure 14:**
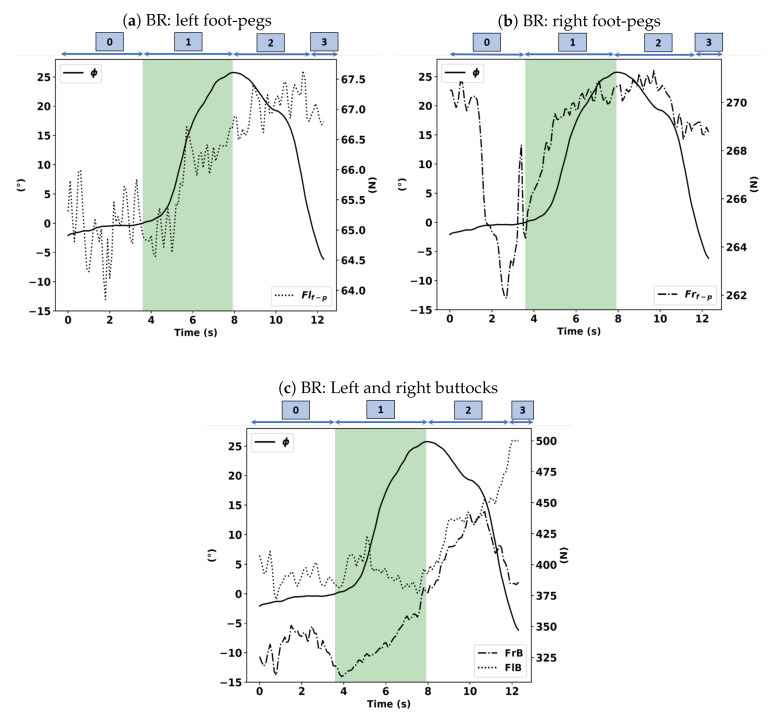
Left and right foot-pegs ($Fl_{f-p}$, $Fr_{f-p}$), buttocks forces (FlB, FrB) expressed in newtons (N) with the roll angle (ϕ) expressed in degrees (${}^{{}^{\circ}}$) in the BR instruction.

In the BR case, as shown in Figure 14a–c, we notice a change in value of the force applied by the driver on the right footrest at t = 3 s before starting phase 1, which corresponds to the instant of the start of the turn. From that moment, this force continuously increases until the end of phase 1 and the beginning of phase 2. In addition, there is no large variation in the force applied by the driver to the left footrest. We also notice the same behavior on the force applied by the right buttock, which implies that simultaneously, the rider is trying to move his/her center of gravity and pressing the right footrest.

**Figure 15:**
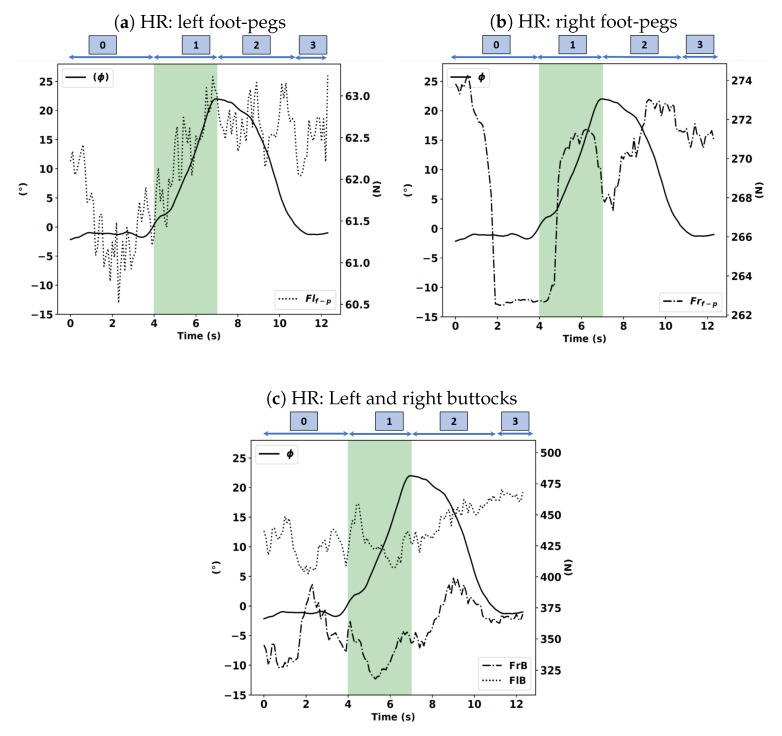
Left and right foot-pegs ($Fl_{f-p}$, $Fr_{f-p}$), buttocks forces (FlB, FrB) expressed in newtons (N) with the roll angle (ϕ) expressed in degrees (${}^{{}^{\circ}}$) in the HR instruction.

In the HR case, as shown in Figure 15a–c, we observe no change in value of the force applied by the driver on the right footrest, and it is even kept constant, and during phase 1, no tendency emerges in its evolution. A similar trend is noted for the force applied by the right buttock evolution. Thus, there is no voluntary action on the driver’s part in this condition.

Thus, we highlighted the relationship between the behavioral representation of the driving practices characterized by sensors and the actions of the rider.

## 6. Conclusions

In this article, the objectives were to use data collected in a real-life experiment to analyze the behavior of several riders during bend taking, identify riders who respect handlebar riding and body riding instructions, and verify those who are consistent with the preferences declared in a questionnaire. We have developed a data mining approach based on feature extraction (using a dimensionality reduction technique) and automatic clustering. Through the standard deviation, the variability of the handlebar is a discriminating feature for rider clustering. The partitioning of provided riders essentially results in two groups according to whether high or low demands are placed on the handlebar during bend riding. Subjects with atypical behaviors were also highlighted. With three riding instructions provided to the riders, we identified the subjects who respect the riding instruction and those whose riding is consistent with the questionnaire. Finally, we initiated an in-depth analysis of bend-taking practices of one subject based on measurements from his inputs (pressures on the handlebar, tank, and footrests). The preliminary results presented in this study are promising. As this work is a part of ongoing research work, future trials should assess this framework methodology’s effectiveness. In particular, additional experiments, including a higher number of subjects, should be considered.

This work calls for several perspectives. The bend-taking analysis in this work considers the curve in its entirety and depends on a parameterization of the signals regarding physical knowledge. A possible alternative is to formalize the problem as automatic segmentation of multivariate time series [39,40]. This segmentation will enable a differentiated and in-depth analysis for the entrance, middle, and end of the bend. Finally, measurements from sensors installed on the rider are included to study the visual anticipation, postural aspects, and their relationship with trajectory control.

## Figures and Tables

**Table 1:** Sensors and their description.

PTW Dynamic Measurements				
**$N^{{}^{{}^{\circ}}}$**	**Sensor**	**Measurement**	**Symbol**	**Description**
1	Two Hall effect sensors	Wheel Speed	*v*	Velocity along the longitudinal axis
2	SICK DT35 Laser [18]	Roll angle measurement	ϕ	Two laser sensors are placed on both sides (right and left) of the motorbike to measure the roll angle of the motorcycle
3	Magnetic sensor AS5047P of AMS [19]	Steering angle	δ	To acquire the handlebar steering angle
4	MTi Xsens [20]	Three-dimensional (accelerometers, magnetometers, and gyroscopes)	−	To acquire inertial movements: longitudinal, lateral, vertical accelerations, and rotational velocities and angles (pitch, yaw, and roll)
**Rider Action Measurements**				
5	Strain gauges [21]	Applied forces on the handlebar	FrX and FlX	Strain gauges are placed on the half-handlebars (right and left) of the motorbike to measure the forces applied by the rider on each half-handlebar (right and left)
6	Mesurex D2 piezoelectric force button [21]	Applied forces on the foot-pegs	$Fr_{f-p}$ and $Fl_{f-p}$	Strain gauges are placed on the (right and left) foot-pegs of the motorbike to measure the forces applied by the rider on each foot-peg
7	XSENSOR LX100 and PX100 pressure matrix pads [22]	Left and right pressure of the buttock	LbP and RbP	To acquire pressure forces of the rider’s buttocks
**Rider Motion Measurements**				
8	Tea Ergo CAPTIV Motion IMU [23]	Roll angle measurement	LbRa	To measure a lower-body roll angle
9	Tea Ergo CAPTIV Motion IMU [23]	Roll angle measurement.	HbRa	To measure a higher-body roll angle.
10	Tea Ergo CAPTIV Motion IMU [23]	Roll angle measurement	−−−	To Mmeasure a head roll angle.
**Context Information**				
11	RTK-GPS Septentrio Altus APS3G [24]	Latitude and longitude positions	GPS	To acquire a precise real-time kinematic positioning of the motorcycle.
12	Video camera	Context videos	−	Action camera on the top case, looking to the front (field of view including the back of the rider).

**Table 2:** Subjects’ declarative data.

Subjects	LA	km	Preference	Instruction Order
S1	2	8000	handlebar	FR, HR, BR
S2	7	3000	handlebar	FR, BR, HR
S3	0	0	body	FR, HR, BR
S4	11	0	body	FR, HR, BR
S5	2	20,000	handlebar	FR, BR, HR
S6	16	0	handlebar	FR, HR, BR
S7	18	2000	handlebar	FR, BR, HR
S8	12	3000	handlebar	FR, HR, BR

**Table 3:** Anderson–Darling test *p*-value for all riding instructions and variables.

Subjects	δ			*v*			*an*			J			C		
	FR	HR	BR	FR	HR	BR	FR	HR	BR	FR	HR	BR	FR	HR	BR
S1	0.69	0.51	0.96	0.99	0.99	0.91	0.99	0.99	0.99	0.70	0.30	0.74	0.99	0.99	0.99
S2	0.97	0.90	0.95	0.99	0.99	0.99	0.99	1	1	**0.24**	0.87	0.99	1	1	0.99
S3	0.96	0.66	0.70	0.99	0.99	0.99	1	0.99	0.99	0.84	0.90	0.92	1	0.99	0.99
S4	0.99	0.98	0.99	01	0.99	0.91	0.99	1	0.99	0.85	0.94	0.99	0.99	0.99	0.99
S5	0.74	0.92	0.99	0.90	0.99	0.99	1	0.99	0.99	0.76	0.92	0.77	1	0.99	1
S6	0.55	0.74	0.81	0.88	0.99	0.99	0.99	0.99	1	0.99	0.64	0.98	0.99	0.99	1
S7	0.99	0.97	0.92	0.99	0.99	0.99	1	01	0.99	0.96	0.85	0.88	0.99	0 1	0.99
S8	0.95	0.98	0.90	0.99	0.94	0.99	1	0.99	0.99	0.48	0.26	0.99	0.99	0.99	0.93

**Table 4:** Clustering results using the Dynamic Time Warping (DTW) and Pearson correlation metrics.

Clusters	Metrics	
	DTW	Pearson Correlation
Cluster 1 (Red)	S3 (FR, HR, BR)	S3 (FR, HR, BR)
Cluster 2 (Green)	S1 (FR, HR, BR)	S1 (FR, HR, BR)
	S2 (FR, HR, BR)	S2 (FR, HR, BR)
	**S4 (FR, BR)**	**S4 (FR, BR)**
	S8 (FR, HR, BR)	S8 (FR, HR, BR)
Cluster 3 (Blue)	**S4 (HR)**	**S4 (HR)**
	S5 (FR, HR, BR)	S5 (FR, HR, BR)
	S6 (FR, HR, BR)	S6 (FR, HR, BR)
	S7 (FR, HR, BR)	S7 (FR, HR, BR)

**Table 5:** *p*-value of the AD test between clusters 2 and 3.

Riding Variables	*p*-Value
Handlebar steering angle	**0.001**
Velocity	0.25
Normal acceleration	0.25
Jerk	0.25
Curvature	**0.001**
